# The Long-Term Course of Outcomes for Lumbar Intervertebral Disc Herniation following Integrated Complementary and Alternative Medicine Inpatient Treatment: A Prospective Observational Study

**DOI:** 10.1155/2017/5239719

**Published:** 2017-08-27

**Authors:** Moon-Hwi Kim, Yoon Jae Lee, Joon-Shik Shin, Jinho Lee, Haechan Jeong, Me-riong Kim, Sam-Min Park, Ung Go, Sung-Moon Kim, Jae-Yeong Kim, Dong-Gyu Hwang, In-Hyuk Ha

**Affiliations:** Jaseng Spine and Joint Research Institute, Jaseng Medical Foundation, Gangnam-gu, Seoul, Republic of Korea

## Abstract

A prospective observational study was conducted in 524 lumbar intervertebral disc herniation (LDH) inpatients to report the long-term effects of complementary and alternative medicine (CAM) treatment. Participants received integrative CAM treatment during hospitalization, from June 2012 to May 2013, and long-term outcomes were assessed from July to August 2016. Numerical rating scales (NRSs) of back and leg pain, the Oswestry disability index (ODI), satisfaction, surgery, recurrence, and current care status were investigated. Baseline characteristics were analyzed to determine factors that predicted long-term satisfaction. A total of 367 patients were available for follow-up. The long-term change in NRS of back and leg pain and ODI was 3.53 (95% CI, 3.22, 3.83), 2.72 (2.34, 3.11), and 32.89 (30.21, 35.57), respectively, showing that improvements were well sustained. Regarding satisfaction, 86.11% responded that they were “slightly improved” or better. Range of lumbar flexion ≤ 60° and both legs' pain at admission were significant predictors of “much improved” or better satisfaction in the long term. Overall, LDH patients who received CAM treatment maintained favorable states in the long term. However, as an uncontrolled observational study, further studies with placebo and/or active controls are warranted.* Trial Registration*. This trial is registered with ClinicalTrials.gov  NCT02257723 (date of registration: October 2, 2014).

## 1. Introduction

Lumbar intervertebral disc herniation (IDH) incurs pain, numbness, and weakness as a result of localized disc displacement beyond the margins of the intervertebral disc space [1]. Low back pain (LBP), which is the main characteristic symptom of lumbar IDH, was reported to be the greatest cause of years lived with disability (YLDs) in the 2010 Global burden of disease, showing that it is a disease that entails considerable personal, social, and economic burden [2]. Recommended treatments for lumbar IDH include surgery and conservative methods such as transforaminal epidural steroid injection [1]. However, injections have been linked with various serious adverse events such as dural puncture and infection [3]. In addition, although early surgical intervention is effective for swifter alleviation of sciatica compared to conservative treatment, the results from 1 year onwards were similar [4], and cases of failed back surgery syndrome where chronic pain persists after surgery continue to be reported [5]. Partly due to these limitations in current conservative and surgical treatments, various complementary and alternative medicine (CAM) treatments are gaining wider recognition and use. The updated 2017 LBP guidelines published by the American College of Physicians recommend such nonpharmacologic treatments as acupuncture and manipulation before administering pharmacologic interventions for chronic LBP, indicating that the effectiveness and safety of acupuncture for LBP are acknowledged and accepted in mainstream conventional medicine. The Korean medical system which employs a dual system of conventional medicine and traditional Korean medicine is unique, and CAM treatment using such traditional methods as acupuncture and herbal medicines for musculoskeletal disorders is commonly used. In Korea, not only is CAM, with focus on traditional Korean medicine therapies, widely used for lumbar IDH treatment, but it is also frequently applied to inpatient care. The Korea National Health Insurance Service claims data show that 22,749 patients were admitted to Korean medicine hospitals under the 6th Korean Standard Classification of Diseases (KCD-6) code M51 (other intervertebral disc disorders) to receive nonsurgical CAM treatment in 2014 [6]. The fact that CAM-related inpatient treatment is widespread in Korea seems to be a digression from the main body of evidence which purports that bed rest is ineffective for lumbar IDH [7]. This high admission rate may be partly due to the fact that CAM-related inpatient treatment is partially covered by private insurance and that hospitalization is mainly for the purpose of receiving concentrated and intensive care as opposed to bed rest. Along these lines, many physicians concurred that inpatient treatment is useful for lumbar IDH in a survey on Korean medicine doctors (KMDs) in Korea [8]. The authors have previously published the short-term results and characteristics of intensive inpatient treatment using CAM [9]. There is, however, a paucity of studies observing the long-term outcomes of inpatient treatment, and the authors therefore assessed and report the long-term results of CAM inpatient treatment.

## 2. Methods

### 2.1. Study Design

This prospective observational study was conducted in patients with radiating leg pain and/or LBP diagnosed with positive MRI findings and KMD diagnosis as lumbar IDH hospitalized at Jaseng Hospital of Korean Medicine during the 1-year period from June 1, 2012, to May 31, 2013. This study investigated the long-term effects of integrative CAM inpatient treatment by following their progress. Jaseng Hospital of Korean Medicine, which is situated in Seoul, Korea, is certified by the Korean Ministry of Health and Welfare as a spine-specialty Korean medicine hospital and accordingly provides advanced CAM treatment for various spine-related disorders. The study participants were treated with herbal medicine, acupuncture, pharmacopuncture, bee venom pharmacopuncture, and Chuna manual therapy for the duration of hospital stay, and further details of study inclusion and exclusion criteria and interventions can be found in the previous publication by Shin et al. [9]. The participants from the Shin et al. study were contacted by phone for long-term follow-up from July to August 2016. The outcome measures assessed at the long-term follow-up are as follows:

- Numerical rating scale (NRS) of LBP and radiating leg pain
- Oswestry disability index (ODI)
- Seven-point scale of satisfaction with current state
- Whether the participant received lumbar surgery after discharge
- Whether the participant was recommended for lumbar surgery prior to hospitalization
- Whether the participant experienced pain recurrence with duration of ≥1 month after conclusion of treatment
- Whether the participant currently suffers from LBP and/or leg pain (attributed to lumbar IDH) for ≥3 months of a level that interferes with daily activities
- Whether the participant currently receives treatment relating to LBP or leg pain.

The 8 items listed above were constructed into structured interviews for the phone surveys. The long-term follow-up study received approval from the Institutional Review Board of Jaseng Hospital of Korean Medicine (JASENG 2016-06-006).

### 2.2. Statistical Analysis

All analyses were performed using statistical package SAS version 9.4 (SAS Institute Inc., Cary, NC, USA), and *p* < 0.05 was considered to be statistically significant. All continuous variables were calculated as mean and standard deviation and categorical variables as frequency and percentages (%). Significance was analyzed using the differences and 95% confidence intervals in average NRS of LBP and radiating leg pain and ODI measured at admission (baseline), discharge, and long-term follow-up. In addition, satisfaction which was initially assessed using a 7-point Likert scale was further dichotomized into satisfied (very satisfied and satisfied) and dissatisfied (slightly satisfied, no change, slightly dissatisfied, dissatisfied, and very dissatisfied) to perform a multivariate logistic regression analysis to determine which factors are predictive of satisfaction at long-term follow-up, and the results are presented as odds ratios (ORs) and 95% confidence intervals (CIs). Variables assessed at baseline which satisfied *p* < 0.10 in univariate analysis were selected and included with age and sex in the final model and analyzed in a stepwise manner (*p* < 0.05).

## 3. Results

**Figure 1:**
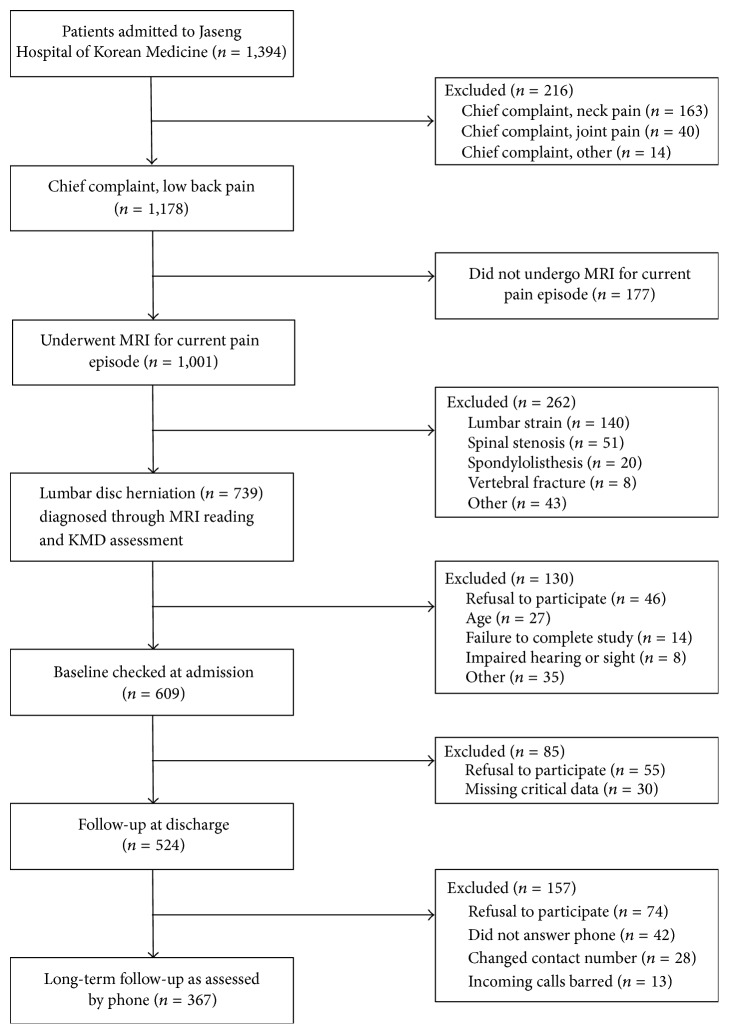
Study flow chart.

A total of 524 patients were followed up for short-term analysis at discharge in the initial study. All participants were contacted for long-term follow-up, of whom 74 refused to participate, 42 did not receive phone calls, 28 could not be contacted due to change in number, and 13 had banned incoming calls or suspended call reception, and were accordingly excluded. Long-term follow-up was conducted in a total 367 participants. The overall flow chart of the study is presented in Figure 1. The participants were analyzed as the long-term follow-up group and non-follow-up group to collectively compare the characteristics of the 2 groups, and the results are presented in Table 1. The non-follow-up group was younger and had a higher female percentage but was otherwise comparable to the long-term follow-up group. The inpatient treatment contents were also compared in the long-term follow-up group and non-follow-up group, but the results did not reveal any substantial differences (Table 2).

The outcome measurements of NRS of LBP, NRS of radiating leg pain, and ODI at admission (which was set as baseline), discharge, and long-term follow-up all showed statistically significant decrease. LBP was reported to be 5.91 ± 2.37 at baseline, which decreased to 2.76 ± 1.89 at discharge, and was maintained at 2.39 ± 2.20 at long-term follow-up. Also, the chief complaint out of LBP and radiating leg pain exhibited improvement in pain over minimal clinically important difference (MCID) (reported as a reduction in NRS of 3.5 in acute pain of ≤4 weeks and 2.5 in chronic pain), and ODI scores also showed functional improvement over the MCID for ODI of 10 (Table 3) [10].

The average period between admission and follow-up was 1316.18 ± 109.56 days. A total of 502 patients had answered the 5-scale satisfaction with current state item at discharge, and 89% had reported satisfaction rates of “satisfied” or higher. A total of 367 participants responded to the 7-scale satisfaction item at long-term follow-up, of whom 86.11% displayed satisfaction rates of “slightly satisfied” or higher and 56.68% “very satisfied” or higher. While 201 (54.77%) out of 367 long-term follow-up participants were confirmed to have been recommended lumbar surgery prior to being admitted at this hospital, only 29 participants (7.90%) went on to receive surgery after inpatient treatment. A total of 121 (32.97%) patients had experienced pain recurrence of 1 month or longer following conclusion of CAM treatment, and 87 (23.71%) reported current LBP and/or leg pain (due to lumbar IDH) severe enough to interfere with daily activities of a duration of 3 months or longer. A total of 99 participants were currently undergoing treatment for LBP and/or leg pain, out of which 48 (13.08%) had opted for CAM treatment, 36 (9.81%) had selected conventional treatment, and 15 (4.09%) were receiving both CAM and conventional treatments (Table 4).

Analysis of predictive factors at admission for satisfaction scores of “very satisfied” or higher at long-term follow-up revealed that range of lumbar flexion ≤ 60° and radiating pain in both legs were significant factors for long-term follow-up satisfaction. Age- and sex-adjusted logistic regression analyses yielded ORs of 2.231 (95% CI 1.030, 4.832) for both leg radiating pain and 0.652 (95% CI 0.426, 0.999) for lumbar flexion of ≤60° (Table 5).

## 4. Discussion

There is no consensus as to which treatment modality is the most effective method of conservative treatment for lumbar IDH. Although epidural steroid injection is the most frequently used conservative treatment in clinical practice, it has only been shown to be successful in achieving short-term pain relief, and its long-term effect on pain and function is unclear [3]. In addition, North American Spine Society (NASS) guidelines state that physical therapy and exercise lack sufficient evidence, which is also the case for a substantial number of pharmacological treatments [1]. It can be surmised that there is no conclusive, undisputed method regarding conservative treatment for disc disorders as of yet.

The authors therefore propose CAM inpatient treatment as an alternative to conventional conservative treatment and accordingly report the short-term and long-term results. The authors previously commented on the fact that the 2011 US Health Cost and Utilization Project database does not seem to give due consideration to conservative inpatient treatment for disc disorders as the majority of inpatients are hospitalized to receive surgery in the US. These results may relate to the previous literature which states bed rest is not effective for sciatica. However, the nature of the CAM inpatient treatment commonly used in Korea is different and is to the aim of providing concentrated CAM treatment rather than bed rest. Consistent with KMD opinion that inpatient care is effective in the early stages of CAM treatment for lumbar IDH, intensive CAM inpatient treatment is currently widely used in Korea. The greatest significance of this study lies in that this is one of the first reports on the long-term effects of CAM inpatient treatment.

These results indicate that inpatient treatment with a focus on CAM for LBP and radiating leg pain patients with lumbar IDH is effective for long-term pain reduction and functional recovery. The follow-up group (*n* = 367) maintained decreased LBP and radiating leg pain intensity compared to admission (baseline) in the long term. Compared to discharge, pain intensity levels declined further, and ODI scores were considerably lower than both admission and discharge. The non-follow-up group (*n* = 157) did not show substantial difference with the long-term follow-up group in terms of treatment contents during the hospitalization period or pain levels at discharge. The MCID of LBP as assessed by NRS has been reported at 2, while the ODI was put at 10 in the previous literature [11], and the present results displayed mean improvement beyond MCID in both pain and functional measures, satisfying the minimal clinical difference in addition to statistical significance at both short-term and long-term follow-ups. However, regarding the difference between discharge and long-term follow-up, while the improvement in ODI scores for functional disability surpassed the MCID of 10, the amount of pain relief was not clinically pronounced though the difference in pain reached statistical significance, and this may be due to the floor effect. Although 121 participants (32.97%) reported pain recurrence and fluctuations following treatment, the number of patients presenting with pain was 87 (23.71%) at the long-term follow-up, displaying a general pattern towards stabilization. A total of 268 participants (73.02%) were currently not receiving treatment for LBP, and 316 (86.11%) responded that they were “slightly satisfied” or higher, while 208 (56.68%) replied that they were “very satisfied” or higher. Though the majority of patients (86.11%) replied that they were satisfied with their current state at long-term follow-up, satisfaction rates were lower than at discharge which may seem strange considering that pain and function scores had improved further in the long term. This may be partially attributed to emotional factors as surgery was recommended for most patients for their condition prior to receiving treatment at this hospital (e.g., relief on having successfully avoided surgery) and as pain and functional disability levels had improved by almost 50% following about 25 days of inpatient care. Meanwhile, despite the fact that functional disability had decreased substantially at 3.6 years compared to discharge, some still suffered mild pain, which may account for the lower satisfaction rate. Additional explanations include divergences in data collection method: the scales used to assess satisfaction levels at discharge and long-term timepoints (5-point versus 7-point scale) were different, and while the outcome assessments at discharge were performed face to face, long-term assessments were conducted by telephone (i.e., the participants may have answered that they were more satisfied when assessed face to face).

The participants were recategorized into responders who showed satisfaction rates of “much improved” or higher and nonresponders to CAM treatment at long-term follow-up in order to determine factors predictive of satisfaction, and the characteristics were analyzed through multivariate logistic regression. Patients with both legs radiating pain tended to be more satisfied with treatment, while patients with restricted range of lumbar flexion presented lower satisfaction rates compared to those without. The reason for the difference in patients with both leg radiating pain may be that as they suffer from neurological symptoms of higher severity, these patients are more likely to experience a greater amount of improvement compared to baseline, potentially heightening satisfaction rates. Radiating leg pain is a considerable obstacle in resuming daily activities and work due to high levels of disability and incurs significant delays in recovery as a consequence. It is therefore commonly referred to as a marker of symptom severity. These results are also consistent with previous studies that report that patients with radiating leg pain, especially those with positive results on neurological tests such as SLR tests, tend to show lower quality of life and greater healthcare use [12–15]. In addition, patients with restricted lumbar flexion may be less satisfied due to various pathological structural changes and dysfunctions secondary to lumbar IDH. Structural change and dysfunction generally impede return to work and daily activities, possibly resulting in lower satisfaction rates from greater levels of restriction. The previous short-term logistic regression results where nonresponders were compared with responders based on MCID improvement are also compliant with these results.

While 54.77% of participants were recommended for surgery prior to admission, only 7.9% were confirmed to have undergone surgery after discharge at the long-term follow-up. Out of the 29 patients who underwent surgery, 12 (41.4%) had been recommended for lumbar surgery prior to hospitalization, and 17 (58.6%) had not. Furthermore, of the 12 patients who had been recommended for and received surgery, only 2 currently suffered from pain levels of ≥NRS 4, whereas of the 17 patients who had not been recommended for but ended up receiving surgery, 7 reported current pain levels of ≥NRS 4 and 2 patients were confirmed to have received 3 surgical operations. It can be inferred that factors other than disc displacement severity are involved in chronic pain development in patients undergoing surgery following CAM inpatient treatment. This is an area that researchers may consider for further exploration in clinical studies of larger sample sizes.

The previous literature was searched for other long-term studies where lumbar IDH patients were followed for ≥3 years to draw relevant comparisons with the current study, and the SPORT study [16] which reported the 4-year results of surgical versus nonsurgical treatment using the ODI for functional disability measurement was compared with the current findings. The collective mean baseline ODI of the surgical and nonsurgical groups in the SPORT study was 49.3 (surgical group baseline ODI: 54.9; nonsurgical group baseline ODI: 38.8) and comparable to the mean baseline ODI of this study (48.21). While the decrease in ODI in the surgical group was 38.1 and that of the nonsurgical group was 24.9 in SPORT, the decrease observed in this study was 32.89, which is about 5.2 less than the surgical group and approximately 8.0 more than the nonsurgical group, indicating that these outcomes are proportionate to previous studies on lumbar IDH treatment.

Limitations of this study include that a sizable proportion of patients were unavailable for long-term follow-up. The non-follow-up group had a slightly higher percentage of women and was slightly younger compared to the long-term follow-up group. Out of 524 patients who were registered at baseline, 367 (70%) were included for long-term follow-up which is not high. The follow-up rate probably reflects the current circumstances of Korean society in addition to population characteristics and general attitudes towards clinical research. The working population is mainly comprised of younger individuals who are generally uninterested and indifferent to clinical study participation due to various preoccupations. This may be partly the reason why those unavailable for long-term follow-up tended to be younger. Also, as commercial telemarketing and spam calls are becoming more prevalent, patients frequently did not pick up follow-up phone calls mistaking them to be telemarketing. As the long-term follow-up was conducted after approximately 3.5 years had lapsed since admission, the contact information from admission was no longer up-to-date, and a number of patients did not answer the phone calls or were otherwise unable to be reached. Another limitation is that this prospective observational study does not have a control group. Lacking a control group, this study is unable to assess whether CAM inpatient treatment is superior to other treatments or natural history. Regarding generalization, the findings of this observational study hold relevance in that the study illustrates actual clinical practice of CAM in hospitalized lumbar IDH patients including treatment type information and their progress. However, this study is limited in that it cannot conclude which CAM treatment type is more effective and/or efficient and that the results cannot be extrapolated to outside of Korea and do not apply to the wide range of CAM modalities not covered in this study. The CAM treatment used in this study was referred to as conservative compared to surgery, a modality commonly employed for treatment of disc disorders. However, while inpatient treatment is widely used in Korea as a means of intensive treatment, 24 days of hospital stay may be viewed as a radical form of treatment in other countries or under different circumstances, and caution need be exercised in interpretation of these results.

Still, this study holds various strengths, especially compared to previous studies on lumbar IDH treatment regarding the following aspects. First, although there are a multitude of studies reporting the effects of surgery, this study specifically focused on nonsurgical treatment using CAM and investigated the effects of inpatient treatment that was not for surgical means. It is not common for patients to be hospitalized unrelated to surgery, and this paper observed the long-term effects of CAM inpatient treatment. The main objective and focus of lumbar IDH treatment should be pain and functional recovery for a safe and swift return to everyday activities, along with absence of long-term symptom recurrence. These results demonstrate that CAM inpatient treatment is able to fulfil these treatment objectives. In addition, CAM inpatient treatment resulted in positive long-term effects and most patients (86.1%) were satisfied with their current state.

## 5. Conclusion

In conclusion, LBP and/or radiating leg pain patients diagnosed with lumbar IDH integrative inpatient treatment with a main focus on CAM exhibited both short-term and long-term clinical improvement in pain and functional scales through intensive inpatient treatment. The majority of patients replied that they were satisfied with their current state.

## Figures and Tables

**Table 1:** Baseline demographic characteristics.

Variables	Long-term follow-up group (*n* = 367)			Non-long-term follow-up group (*n* = 157)		
	*n*	%	Mean (SD)	*n*	%	Mean (SD)
Age (years)			43.72 ± 13.07			38.33 ± 12.65
Gender						
Male	199	54.22		67	42.68	
Female	168	45.78		90	57.32	
Smoking status						
No	247	67.30		86	54.78	
Quit	63	17.17		26	16.56	
Yes	57	15.53		45	28.66	
Drinking						
Yes	301	82.02		139	88.54	
No	66	17.98		18	11.46	
Body Mass Index (kg/m^2^)			23.96 ± 8.29			23.49 ± 3.44
Previous back pain						
None	243	66.21		101	64.33	
Disc herniation	109	29.70		46	29.30	
Other (e.g., fracture)	15	4.09		10	6.37	
Comorbid illnesses^a^						
Yes	61	16.62		16	10.19	
No	306	83.38		141	89.81	
Radiating pain						
None	79	21.53		24	15.29	
Unilateral	226	61.58		99	63.06	
Bilateral	62	16.89		34	21.66	
Radiating pain to below the knee						
None	136	37.06		60	38.22	
Unilateral	225	61.31		95	60.51	
Bilateral	6	1.63		2	1.27	
Muscular weakness	47	12.81		21	13.38	
Sensory loss	42	11.44		15	9.55	
Straight leg raising test < 60°	112	30.52	38.68 ± 9.71	63	40.13	40.63 ± 8.87
Range of lumbar flexion < 60°	138	37.60	27.15 ± 15.68	62	39.49	24.79 ± 16.34
Previous back surgery	34	9.26		15	9.55	
Previous treatment (for current pain episode)						
Nerve blocks	107	29.16		42	26.75	
Pain killers	108	29.43		35	22.29	
CAM	70	19.07		28	17.83	
Duration of current episode (days)						
Less than 1 month	144	39.24		61	38.85	
Between 1 and 6 months	117	31.88		45	28.66	
Other	106	28.88		51	32.48	
Low back pain, NRS (0–10)			5.91 ± 2.37			6.22 ± 2.45
Radiating leg pain, NRS (0–10)			5.11 ± 3.11			5.23 ± 3.24
Oswestry disability index (0–100)			48.21 ± 20.60			49.51 ± 21.11

NRS, numerical rating scale; CAM, complementary and alternative medicine; ^a^any self-reported gastritis, tuberculosis poliomyelitis, cardiovascular diseases, uterine myoma, or hepatitis B carrier.

**Table 2:** Length of hospital stay and interventions administered during stay.

Variables	Long-term follow-up group (*n* = 367)				Non-long-term follow-up group (*n* = 157)			
	*n*	%	Mean	SD	*n*	%	Mean	SD
Length of hospital stays (days)			24.90 ± 13.37				23.13 ± 12.82	
Complementary and alternative medicine								
Herbal medicine^a^	366	99.73	44.24	24.01	157	100.00	40.62	20.55
Protocol decoction	353	96.19	22.56	12.92	153	97.45	21.52	12.65
Protocol pills	366	99.73	48.01	29.05	157	100.00	43.23	23.79
Acupuncture	367	100.00	37.77	21.47	157	100.00	35.03	21.05
Pharmacopuncture	323	88.01	15.34	13.09	132	84.08	14.33	13.47
Bee venom pharmacopuncture	190	51.77	19.31	14.15	73	46.50	20.12	11.53
Chuna (Korean manipulation)	300	81.74	13.84	8.54	139	88.54	12.72	7.58
Conventional treatment								
Analgesics (pain killers)	58	15.80	1.4	0.9	31	19.75	1.58	1.15
Nerve blocks^b^	37	10.08	1.08	0.28	22	14.01	1.05	0.21

^a^Herbal medicine protocol: a standardized herbal medicine prescription was recommended for all patients prior to commencement of the study; however, the protocol allowed for individual tailoring according to patient characteristics and clinical symptoms as seen necessary by KMDs; ^b^medications such as anesthetics and steroids were injected in close proximity to the lumbar nerve root affected by the herniated disc.

**Table 3:** Comparison of NRS for LBP and ODI scores in the long-term follow-up group and non-long-term follow-up group.

	Long-term follow-up group (*n* = 367)			Non-long-term follow-up group (*n* = 157)	
	Baseline	Discharge	Long-term follow-up	Baseline	Discharge
Low back pain, NRS	5.91 ± 2.37	2.76 ± 1.89	2.39 ± 2.20^*∗*^	6.22 ± 2.45	2.97 ± 1.86
Diff (95% CI)^a^		3.16 (2.92, 3.39)	3.53 (3.22, 3.83)		3.25 (2.88, 3.61)
Radiating leg pain, NRS	5.11 ± 3.11	2.47 ± 2.06	1.85 ± 2.30^*∗∗*^	5.23 ± 3.24	2.71 ± 2.16
Diff (95% CI)^a^		2.36 (2.00, 2.71)	2.72 (2.34, 3.11)		2.26 (1.72, 2.80)
Oswestry disability index	48.21 ± 20.60	29.27 ± 15.20	15.36 ± 15.87^*∗∗*^	49.51 ± 21.11	28.87 ± 14.57
Diff (95% CI)^a^		18.64 (16.93, 20.94)	32.89 (30.21, 35.57)		20.65 (17.57, 23.73)

NRS, numerical rating scale; ODI, Oswestry disability index; ^a^difference from baseline (95% confidence interval); ^*∗*^*p* < 0.01 in paired *t*-test between discharge and long-term follow-up; ^*∗∗*^*p* < 0.001 in paired *t*-test between discharge and long-term follow-up.

**Table 4:** Long-term follow-up results.

Variables	*n*	%	Mean (SD)
Period from admission date to long-term follow-up (days)			1316.18 ± 109.56
Period from admission date to long-term follow-up (years)			3.61 ± 0.30
Period from admission date to long-term follow-up (months)			43.27 ± 3.60
Experience of surgery^a^ after discharge			
No	337	91.83	
Yes	29	7.90	
Missing	1	0.27	
Recommendation for surgery at another institution			
No	157	42.78	
Yes	201	54.77	
Others	9	2.45	
Previous recurred pain ≥ 1 month after discharge			
No	240	65.40	
Yes	121	32.97	
Others	6	1.63	
Current pain ≥ 3 months			
No	280	76.29	
Yes	87	23.71	
Current treatment^b^			
None	268	73.02	
CAM	48	13.08	
Conventional treatment	36	9.81	
CAM + conventional treatment	15	4.09	
5-scale satisfaction level at discharge			
Very satisfied	177	35.26	
Satisfied	270	53.79	
Slightly satisfied	47	9.36	
Dissatisfied	7	1.39	
Very dissatisfied	1	0.20	
7-scale satisfaction level at long-term follow-up			
Very satisfied	51	13.90	
Satisfied	157	42.78	
Slightly satisfied	108	29.43	
No change	35	9.54	
Slightly dissatisfied	13	3.54	
Dissatisfied	—	—	
Very dissatisfied	2	0.54	
Missing	1	0.27	

CAM, complementary and alternative medicine; ^a^Experience of surgery referred to lumbar operations received between discharge and long-term follow-up; ^b^current treatment was assessed as treatments received within a week previous to long-term follow-up, and types were recategorized into CAM and conventional treatments.

**Table 5:** Assessment of predictive factors at baseline associated with satisfaction rate.

	Univariate		Multivariate	
	OR	95% CI	OR	95% CI
Age (continuous)	1.006	0.993 1.020		
Gender, male (ref. female)	0.921	0.649 1.307		
Smoking status (ref. nonsmoking)				
Quit	0.759	0.474 1.216		
Yes	1.189	0.749 1.887		
Drinking (ref. no)	1.151	0.710 1.864		
Body Mass Index (kg/m^2^) (continuous)	1.009	0.979 1.039		
Previous back pain (ref. no pain)				
Disc herniation	1.429	0.964 2.118		
Other (e.g., fracture)	3.056	1.121 8.332		
Comorbid illness (ref. no comorbidity)^a^	1.781	1.050 3.023		
Radiating pain (ref. no pain)				
Unilateral	1.040	0.666 1.625	0.855	0.511 1.431
Bilateral	3.067	1.638 5.743	2.231	1.030 4.832
Radiating pain below knee (ref. no pain)				
Unilateral	1.076	0.749 1.546		
Bilateral	1.149	0.267 4.947		
Straight leg raising test < 60° (ref. ≧ 60°)	1.017	0.701 1.474		
Range of lumbar flexion < 60° (ref. ≧ 60°)	0.612	0.427 0.877	0.652	0.426 0.999
Surgery (ref. no surgery)	0.866	0.478 1.570		
Previous treatment (for current episode)				
Nerve blocks (ref. no)	0.797	0.542 1.171		
Pain killers (ref. no)	0.693	0.470 1.023		
CAM (ref. no)	0.694	0.446 1.080		
Duration of current episode (days) (ref. < 1 month)				
Between 1 and 6 months	1.082	0.713 1.641		

OR, odds ratio; CAM, complementary and alternative medicine; only statistically significant variables from univariate regression were included using stepwise method in multivariable logistic regression with age and gender (*p* < 0.05); ^a^any self-reported gastritis, tuberculosis, poliomyelitis, cardiovascular disease, uterine myoma, or hepatitis B carrier.
